# Supplementary material for: Systematic comparison of risky choices in humans and monkeys
Source: bioRxiv. 2023 Feb 8:2023.02.07.527517. Preprint. [Version 1] doi: 10.1101/2023.02.07.527517 (PMC9934584; doi:10.1101/2023.02.07.527517)
Supplement: Supplement 1 [file NIHPP2023.02.07.527517v1-supplement-1.pdf]

bioRxiv preprint doi: https://doi.org/10.1101/2023.02.07.527517; this version posted February 8, 2023. The copyright holder for this preprint (which was not certified by peer review) is the author/funder, who has granted bioRxiv a license to display the preprint in perpetuity. It is made available under aCC-BY 4.0 International license.

Supplementary figures

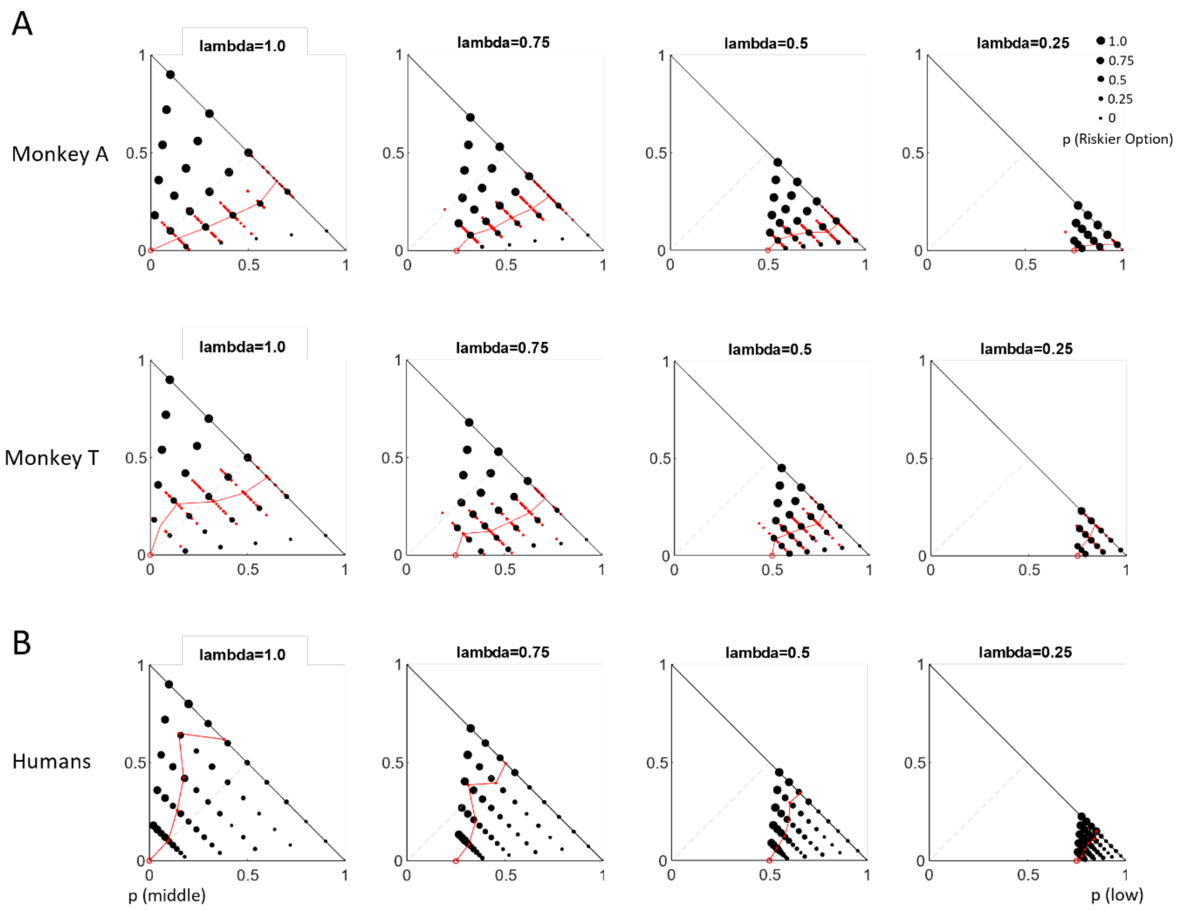

Figure S1. Differential risk attitude across probabilities in the Marschak-Machina triangle.

(A) Choices in the two monkeys: 34 sessions for Monkey A and 26 sessions for Monkey T.

(B) Choices in the 126 human participants: four safer options ($10, p = 1; $10, p = 0.75; $10, p = 0.5; $10, p = 0.25).

Each panel shows the probability of choosing a risky option. Each trial offered two options, a safer option (blue solid dot) and a riskier option (one of the black solid dots). We tested four safer options (0.25ml, p=1; 0.25ml, p = 0.75; 0.25ml, p = 0.5; 0.25ml, p = 0.25), as represented by the four Marschak-Machina triangles (lambda = 1.0, lambda = 0.75, lambda = 0.5, and lambda = 0.25). The probability of choosing the risky option across sessions is represented by the size of the black solid dot. Indifferent points (IP) in each session, indicating p = 0.5 of choosing the risky option (as estimated by softmax function), are represented by red dots. Red hollow circles represent average IPs across all sessions; blue dots represent the safer options.

bioRxiv preprint doi: https://doi.org/10.1101/2023.02.07.527517; this version posted February 8, 2023. The copyright holder for this preprint (which was not certified by peer review) is the author/funder, who has granted bioRxiv a license to display the preprint in perpetuity. It is made available under aCC-BY 4.0 International license.

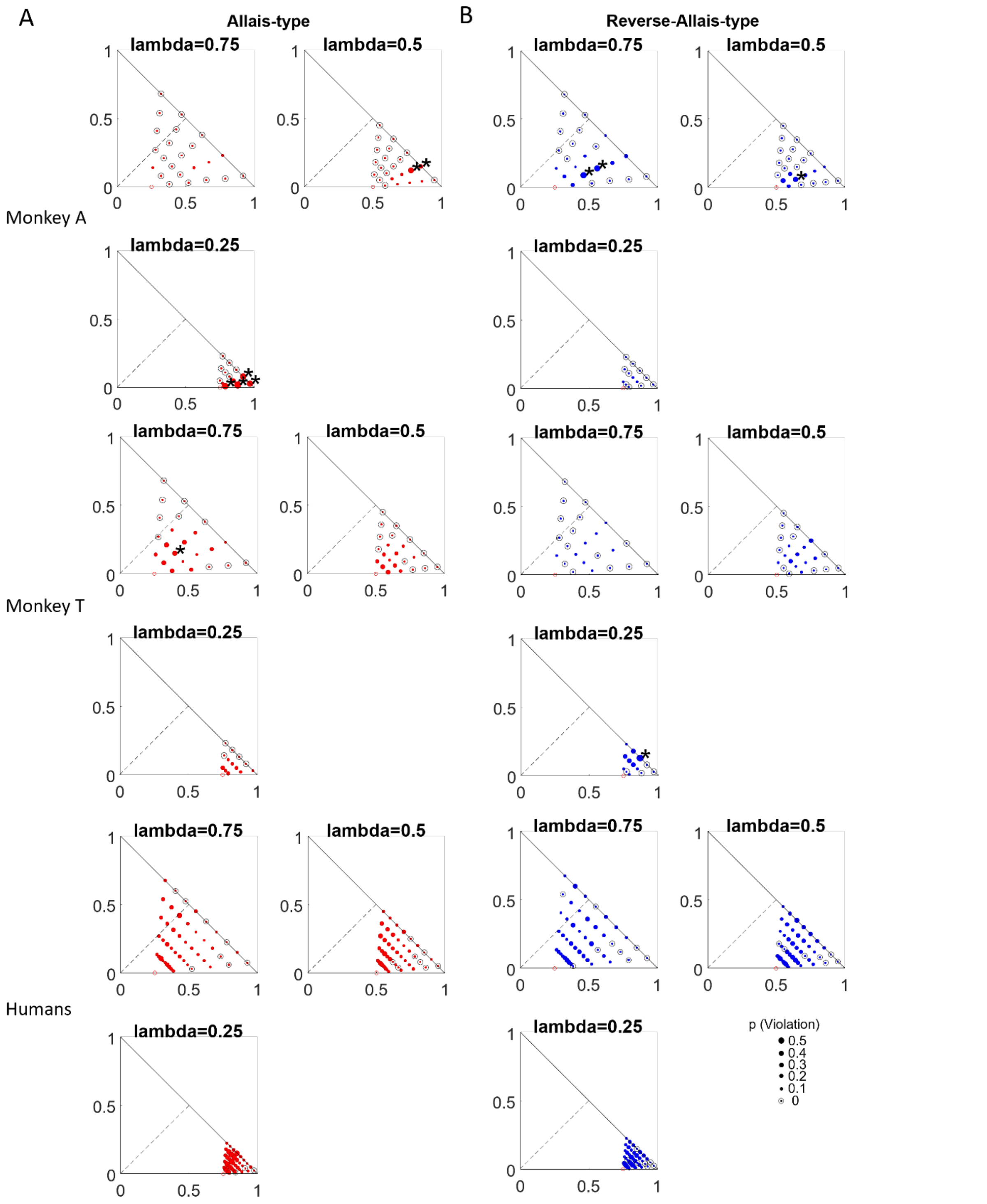

Figure S2. Violations of independence axiom across probabilities

Frequency and direction of the independence axiom violation across different probabilities. Dot size represents the probability of violation across (monkey) sessions or (human) participants, comparing different lambdas to the initial choice between options A and B (lambda = 1.0). (A) Red dots represent the probability of Allais-type violations. (B) Blue dots represent the probability of reverse-Allais-type violations. Hallow black circles indicate no violation for that choice set. * significance $P < 0.05$; binomial test in the two test sets. The correlation between predicted and actual violation was significant (GLM model with human data to predict monkey behavior, Spearman Rho = 0.201, $P = 0.029$), when lambda was used as the regressor.

bioRxiv preprint doi: https://doi.org/10.1101/2023.02.07.527517; this version posted February 8, 2023. The copyright holder for this preprint (which was not certified by peer review) is the author/funder, who has granted bioRxiv a license to display the preprint in perpetuity. It is made available under aCC-BY 4.0 International license.

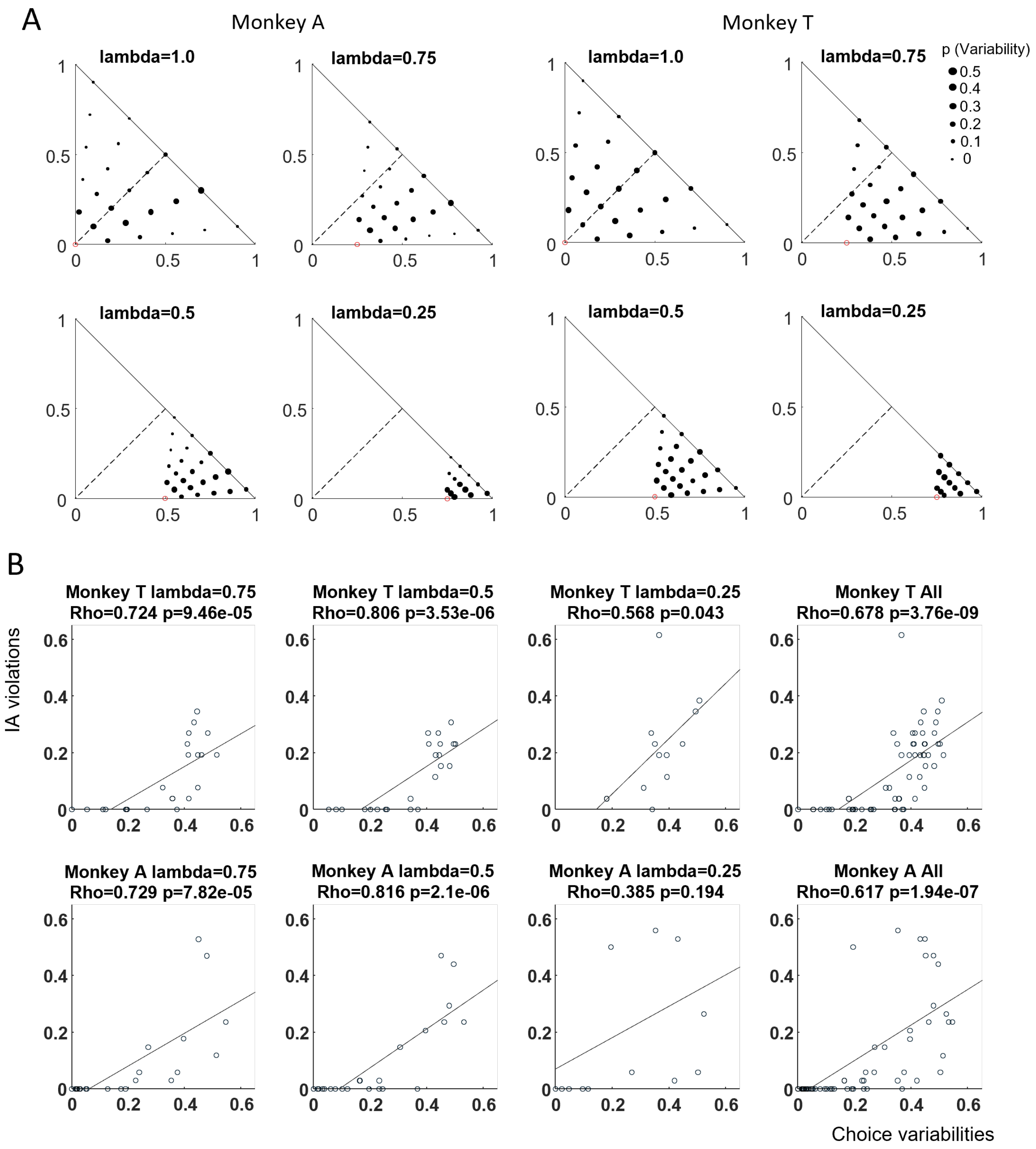

Figure S3. Choice variability partly explains independence axiom (IA) violations in monkeys.

(A) Choice variability (standard deviation) across different probabilities. Size of dots representing the variability (standard deviation) of choices averaged across sessions (34 sessions for Monkey A and 26 sessions for Monkey T).

(B) Correlation of choice variabilities (sum of two tests) and IA violations.
